# The Use of Forecast Accuracy Indicators to Improve Planning Quality: Insights from a Case Study

**DOI:** 10.1080/09638180.2019.1577150

**Published:** 2019-03-05

**Authors:** Silvia Jordan, Martin Messner

**Affiliations:** Faculty of Business and Management, University of Innsbruck, Innsbruck, Austria

## Abstract

Accounting studies have analyzed rolling forecasts and similar dynamic approaches to planning as a way to improve the quality of planning. We complement this research by investigating an alternative (complementary) way to improve planning quality, i.e. the use of forecast accuracy indicators as a results control mechanism. Our study particularly explores the practical challenges that might emerge when firms use a performance measure for forecast accuracy. We examine such challenges by means of an in-depth case study of a manufacturing firm that started to monitor sales forecast accuracy. Drawing from interviews, meeting observations and written documentation, we highlight two possible concerns with the use of forecast accuracy: concerns related to the limited degree of controllability of the performance measure and concerns with its goal congruence. We illustrate how organizational actors experienced these challenges and how they adapted their approach to forecast accuracy in response to them. Our empirical observations do not only shed light on the possibilities and challenges pertaining to the use of forecast accuracy as a performance measure; they also improve our understanding of how specific qualities of performance measures apply to ‘truth-inducing’ indicators, and how the particular organizational and market context can shape the quality of performance measures more generally.

It’s a forecast; the only sure thing about a forecast is that it’s going to be wrong.

(Materials manager at PowerCo)

## 1. Introduction

Planning is a key managerial task (Fayol, 1916; Koontz & O'Donnell, 1955; Taylor, 1911), and many firms invest quite some time and effort in establishing plans and monitoring their realization. One role of plans is to facilitate operational decisions, for instance in the areas of production, inventory management, purchasing, or hiring. Plans may be more or less helpful for such decision-facilitation and concerns with the quality of plans in this respect exist among both practitioners and researchers.

Especially in a context of high uncertainty, planning information can quickly become outdated. To avoid such a loss of planning quality, it has been suggested that firms replace or complement static plans (such as annual budgets) with more ‘dynamic’ forms of planning such as rolling forecasts (Hope & Fraser, 2003). These feature rather short time intervals in order to incorporate upcoming information in a timely way (Lamoreaux, 2011; Morlidge & Player, 2010). Moreover, they often rely on frequent interaction between managers from different departments so as to integrate different functional views (Abernethy & Lillis, 1995). The accounting literature provides several case studies which demonstrate the usefulness of such dynamic and interactive forms of planning to improve planning quality (e.g. Bourmistrov & Kaarbøe, 2013; Goretzki & Messner, 2016; Henttu-Aho & Järvinen, 2013; Østergren & Stensaker, 2010).

While dynamic and interactive forms of planning seek to increase planning quality by means of increasing the timeliness of information, an alternative (complementary) route to increasing the quality of plans is to *incentivize* actors to plan more accurately. Such incentives particularly exist for sales forecasts and they assume the form of performance measures for forecast accuracy. However, compared to the literature on dynamic and interactive forms of planning, we have little knowledge on how firms actually use such performance indicators. Scholars in accounting and operations research have repeatedly suggested forecast accuracy indicators as a remedy to the problem of inaccurate forecasts (e.g. Chow, Hirst, & Shields, 1994; Church, Hannan, & Kuang, 2012; Davis & Mentzer, 2007; Fildes et al., 2003; Helms, Ettkin, & Chapman, 2000), but have not studied the use of such performance measures in practice.

In this paper, we investigate the use of forecast accuracy indicators to incentivize more accurate forecasting inside the firm and across the firm’s boundaries. We draw upon an in-depth case study of a manufacturing organization (PowerCo^1^) that started to systematically monitor sales forecast accuracy after having introduced a new planning and forecasting process. We trace key events in the firm’s concern with forecast accuracy and highlight the challenges that the firm encountered in the course of using this performance measure.

Our findings suggest that, although forecast accuracy was deemed important for PowerCo, it was at the same time experienced as a somewhat problematic performance measure because of its limited degree of controllability and because of concerns with its goal congruence. We show how these concerns were experienced in our case company and we discuss the organizational and market conditions that facilitated their emergence. We also illustrate how organizational actors sought to deal with these challenges, by using forecast accuracy indicators in more interactive ways. Besides these findings on the possibilities and challenges pertaining to the use of forecast accuracy as a performance measure, our empirical observations also allow us to critically discuss the notion of ‘truth-inducing’ performance measures with regard to their typically assumed qualities of controllability and goal congruence. Furthermore, by illustrating the impact of specific inter-organizational relations and of socio-political factors on the controllability and goal congruence of forecast accuracy, our study improves our understanding of how the particular organizational and market context can shape the quality of performance measures more generally. Our paper therefore contributes to the literatures on dynamic planning, on truth-inducing indicators, and on performance measures and their qualities (e.g. Hansen, 2010; Jordan & Messner, 2012; Lillis, 2002; Merchant, 2006).

The structure of our paper is as follows. In the next section, we review existing evidence on forecast accuracy. The following section provides the analytical framework for studying forecast accuracy as a performance measure. Section four explains the research setting and design. Thereafter, we present our empirical findings. Section six offers a discussion of the findings and the final section concludes.

## 2. Literature Review

### 2.1. Planning and Forecast Accuracy

In the accounting literature, planning is typically associated with a motivational and a decision-facilitating role, respectively (Demski & Feltham, 1976). On the one hand, plans reflect performance expectations (often combined with incentives) that are supposed to motivate employees to invest particular effort into the realization of these plans. On the other hand, plans also provide information that helps managers coordinate their activities and make a wide range of operational decisions (Merchant & Van der Stede, 2007).

While firms often use annual budgets to set targets and motivate employees, they deploy more frequently established forecasts to facilitate operational decisions (Sivabalan, Booth, Malmi, & Brown, 2009). These forecasts exist for all kinds of financial or operational items, but of particular relevance in both operational and financial terms are sales forecasts. Sales forecasts provide an outlook on future revenues (and thus allowable costs) and are crucial for coordinating between demand and supply activities. The accuracy of sales forecasts (in terms of correctly predicting future states) is one important component of planning quality, and accurate forecasting is an objective that is high on the agenda of many CFOs and operations managers alike (O'Mahony & Lyon, 2016).

To assess the accuracy of forecasts, firms can resort to various types of forecast accuracy indicators. One widely used indicator of forecast accuracy is the inverse value of the mean absolute percentage error, or MAPE (Fildes & Goodwin, 2007). MAPE represents the mean value of the absolute percentage differences between forecasted values and actual values. If, for instance, forecasted sales for product A are 50 units and actual sales are 100 units (i.e. 50% absolute error), while for product B, they are 220 and 200 units, respectively (i.e. 10% absolute error), then the MAPE would be 30%. Accordingly, the aggregate forecast accuracy in this case would be 70%.

Like other types of performance measures, forecast accuracy indicators can be used as management control tools not only to monitor forecasting quality, but also to incentivize more accurate forecasting by employees or third parties. In the following, we review literature in operations management and accounting, respectively, that has been concerned with such a use of forecast accuracy.

### 2.2. Forecast Accuracy in Operations Management Research

The dominant concern in the operations management literature has been with the technical dimensions of forecasting, in the sense that researchers have used measures of forecast accuracy to assess the relative quality of different forecasting techniques. Much less attention has been given to the managerial uses of forecast accuracy. Those studies that take a more managerial perspective tend to agree on the importance of monitoring forecast accuracy (e.g. Armstrong & Fildes, 2006; Davis & Mentzer, 2007). They assert that firms *should* be concerned with accurate forecasts in order to ensure effective cross-functional and inter-organizational planning and coordination. Forecast accuracy measures can be helpful tools in this respect insofar as they can alleviate problems of motivation and knowledge. Davis and Mentzer (2007), for instance, state that forecast accuracy measurement is important as a feedback mechanism in order to facilitate learning about forecast errors and improve the forecasting process accordingly. Stewart (in Fildes et al., 2003) suggests that team rewards based on accurate forecasts may remedy the problem of biased forecasts that is often caused by individual rewards for beating the forecast. For the case of inter-organizational collaboration across the supply chain, Helms et al. (2000) argue that in order to ‘ensure that forecast accuracy and related supply chain performance actually do improve as a result of the collaborative process, measurement and incentives must be part of the process’ (Helms et al., 2000, p. 400).

While such suggestions to implement forecast accuracy indicators as a remedy to incentive and knowledge problems seem reasonable, there is a lack of empirical accounts of how firms actually use forecast accuracy as a performance measure and of the potential challenges that they thereby face. Some authors have suggested that forecast accuracy indicators may not be unproblematic as they are not always strongly correlated with outcome measures such as inventory performance or customer service levels (Ali, Boylan, & Syntetos, 2012; Goodwin, 2009) or because it can be difficult to define meaningful accuracy targets (Bunn & Taylor, 2001; Davis & Mentzer, 2007). Yet, evidence on such challenges is not extensive and we have little in-depth understanding of how such challenges are experienced and dealt with by managers.

### 2.3. Forecast Accuracy in Accounting Research

Research within accounting has also emphasized the importance of producing accurate forecasts. Accounting studies have looked at forecast accuracy mainly from the perspective of the motivational rather than the decision-facilitating function of plans. Accordingly, the main concern in this literature has been on problems of motivation that appear in the form of intentional misreporting of forecast numbers or so-called ‘budgetary biasing’. A budgetary bias is ‘a deliberately created difference between the budgeting actor’s forecast about the future (‘honest budget estimate’), and his or her submitted budget figure (budget proposal)’ (Lukka, 1988, p. 282). Budgetary biases can be motivated by the intention to establish easily achievable standards for performance evaluation (budgetary slack leading to under-forecasting), by efforts to obtain more resources (upward-bias leading to over-forecasting), or by the desire to leave room for innovation (Davila & Wouters, 2005; Lukka, 1988). Most research has focused on the first two ‘problematic’ types of biases. Some studies have addressed possible solutions to the problem of over- or under-forecasting. They focus on the optimal design of an incentive contract so as to avoid deliberate misrepresentation in forecasts and build upon either analytical (e.g. Ijiri, Kinard, & Putney, 1968; Miller & Thornton, 1978; Osband & Reichelstein, 1985; Weitzman, 1976) or experimental methods (e.g. Brüggen & Luft, 2011; Chow et al., 1994; Church et al., 2012; Evans, Hannan, Krishan, & Moser, 2001; Hannan, Rankin, & Towry, 2006; Waller, 1988). The key message of these studies is that actors responsible for providing forecast information should be incentivized for truthful reporting, i.e. for forecast accuracy. Although insightful, these studies do not show how truth-inducing schemes are used in practice. In light of this, Chow et al. (1994) suggest that efforts should be made to empirically examine ‘the nature of incentive systems found in practice’ (p. 716). This is deemed particularly relevant since sophisticated truth-inducing reward systems developed by the analytical literature are rarely used in practice, as firms tend to prefer relatively simple incentive systems (Baker, Jensen, & Murphy, 1988).

Two observations stand out from the above literature review. First, there seems to be a general lack of empirical research on how forecast accuracy is used in practice and, more specifically, on how it is mobilized as a performance measurement instrument. As Winklhofer observes, ‘we seem to know a great deal about the technical side of forecasting but very little about managing forecasting-related activities and their uses’ (Winklhofer in Fildes et al., 2003, p. 35). Second, analytical and experimental work in accounting frames the managerial challenge of creating accurate forecasts mainly in terms of a problem of motivation that is linked to the phenomenon of budgetary biasing.^2^ In other words, the literature emphasizes the need to incentivize managers for accurate forecasting, assuming that managers indeed have the relevant private information that allows them to provide accurate forecasts. More generally, the very terminology of ‘truth-inducing’ indicators, which seek to measure as much the quality of the plan as the quality of the action performed to achieve the plan, assumes that such performance measures are highly controllable and goal congruent. On the one hand, achieving these performance measures is seen to be a matter of ‘telling the truth’ and is thus regarded as controllable by the person or organizational unit who compiles the plans and forecasts. This assumption is based on the typical agency theoretical framing, where subordinate managers are seen to be better informed than their superiors and need to be incentivized for truthful upward reporting (e.g. Chow et al., 1994, p. 700; Church et al., 2012, p. 155). While some authors (e.g. Evans et al., 2001; Salterio & Webb, 2006) have challenged the assumption that individuals have a general propensity to lie, the basic idea of well informed ‘agents’ who may or may not bias the information provided to under-informed ‘principals’ is generally taken-for-granted in this literature. This pronounced information asymmetry derives from the conceptualization of principal-agent relationships in terms of isolated, hierarchical relations between two actors with clearly differentiated roles. Following this idea, experimental studies on truth-inducing indicators and budgetary slack typically stage the principal-agent relationship as a hierarchical intra-organizational relation between ‘managers’ who are accountable to ‘owners’ (or superiors). More complex relationships, where different hierarchies, actors and accountabilities interrelate, as is typically the case in organizational, and specifically in inter-organizational settings, have not been considered in this literature so far. On the other hand, pursuing truth-inducing indicators is regarded as goal congruent since accuracy of plans reduces budgetary biases and thus ensures that resources are allocated to their most profitable uses. As, for instance, Church et al. (2012, p. 155) state, ‘To the extent that budgetary slack results in unnecessary expropriation of resources by the subunit manager, it is not in the best interest of the overall organization’. More specifically, measures of forecast accuracy are seen to be in line with relevant organizational objectives such as reducing cost of capital (by minimizing stock) and improving customer satisfaction (by increasing on-time delivery). While it makes intuitively sense to assume truth-inducing accuracy indicators to be controllable and goal congruent, we do not know in which way and to what extent these qualities indeed materialize in practice. In the following section, we explain the concepts of controllability and goal congruence in more detail and we place them in the context of the broader analytical lens of *situated qualities* of performance indicators.

## 3. Analytical Lens: Situated Qualities of Performance Measures

One way of making sense of the potential challenges that can emerge with the use of a performance measure is to consider the *qualities* that performance measures should have when they are used to control and influence managerial work. If some of these qualities are not or only marginally fulfilled, there would seem to be a higher probability that managerial problems materialize.^3^ Merchant (2006) lists the following qualities that performance measures should have when being used to motivate and control managerial action: goal congruence, controllability, timeliness, accuracy, understandability, and cost effectiveness. While one can certainly think of more criteria than these, Merchant’s list arguably captures a good deal of what managers will be concerned about when implementing and using performance measures.

The literature has particularly focused on the first two criteria, i.e. goal congruence and controllability. Goal congruence is high when a performance measure is in line with the organization’s objective such that an improvement of the measure translates into an improvement of overall performance. Goal congruence is low if a performance measure only correlates weakly with the overall objective and/or if it represents only part of this overall objective, i.e. if variations in the measure explain only part of the variation in overall performance (Merchant, 2006). A lack of goal congruence can create challenges or problems insofar as managers would be motivated to undertake actions that are not in line with the organization’s objective.

While high goal congruence is generally desirable, it may conflict with a second attribute that is typically considered important for an indicator, i.e. controllability. Controllability refers to the extent to which a manager can influence the value of the performance measure, such that the performance measure reflects the managers’ efforts rather than uncontrollable noise (Antle & Demski, 1988; Hirst, 1983; McNally, 1980). Controllability may conflict with goal congruence, since measures that are highly goal congruent (such as total shareholder return) usually suffer from problems of controllability, while measures that are more easily controllable (such as operational indicators) have a more ambiguous relationship to the overall objective of the organization (Feltham & Xie, 1994). A lack of sufficient controllability is likely to be perceived as problematic by managers as it makes them responsible for something that they cannot influence (e.g. Hopwood, 1972).

The other qualities mentioned by Merchant (2006) have received less attention in the accounting literature. Timeliness refers to the time lag between the manager’s actions and the feedback provided in the form of performance measurement. Timely feedback is typically considered important for motivational reasons, and a lack of timeliness may create problems in the sense that managers cannot learn from their evaluation. Accuracy denotes the precision and objectivity with which performance is measured (Merchant, 2006). Performance measures may be contested when they lack precision and objectivity, as employees may feel that their evaluations do not properly reflect their work results. Understandability means that managers understand how the performance measure works and what they have to do in order to influence it. Again, it is apparent that a lack of understandability is likely to be experienced as problematic by managers who try to influence the measure upon which they are evaluated. Finally, performance measures should also be cost effective to produce such that the informational benefits that the measure provides outweigh the costs of its measurement (Merchant, 2006).

In order to analyse the perceived challenges of forecast accuracy as a performance measure, we conceive of the above outlined qualities not as ‘intrinsic’ features of performance measures, but rather as qualities that are formed in a particular managerial and organizational context in which a measure is used (see Hopwood, 1983). The functionality of performance measures is to be regarded as a *situated* functionality (Ahrens & Chapman, 2007; Goretzki, Mack, Messner, & Weber, 2018) that is not necessarily stable across time and space, but is subject to the characteristics of the organization and its environment. As a consequence, we suggest that also the qualities of performance measures are situated. For instance, whether a performance measure is goal congruent or not obviously depends on how the organization has specified its overall objective. And there may well be differences in opinion in this respect, such that some actors may experience a performance measure to be goal congruent while others don’t. Moreover, individual performance measures will typically capture only part of what is important to a firm. Such incompleteness (Jordan & Messner, 2012; Lillis, 2002) can motivate the use of a combination of measures which, however, may suggest competing courses of action. The situated functionality of one measure is then subject to its (negative) influence on other measures also deemed important (Hansen, 2010). Controllability is equally a function of the managerial and organizational context in place. Whether a performance measure is controllable or not is not just a question of its design characteristics. It also depends on the abilities of the actors who are supposed to be motivated by the performance measure. While some actors may find it easy to influence a performance measure, others will struggle doing so (Burkert, Fischer, & Schäffer, 2011). Technology can also influence the qualities of performance measures. Questions of measurement accuracy and timeliness, for instance, do not only depend on the conceptualization of a performance measure, but also on the technological solutions used for its measurement.

The analytical lens of the situated qualities of performance measures will help us explore in the empirical part of the paper the situated conditions under which the use of forecast accuracy as a performance measure can lead to managerial challenges related to any of these qualities, and how these qualities apply to truth-inducing performance indicators more broadly.

## 4. Research Setting and Methods

Our analysis draws from data collected through a qualitative field study conducted in a single organization. The organization, hereafter called PowerCo, is one of several business units of a Fortune 500 firm that provides technology-intensive products mainly for public and industrial customers (hereafter referred to as ParentCo). At the time of our field work, the company employed around 40,000 people and generated annual revenues of more than $10bn, of which PowerCo realized around 20%. Corporate headquarters as well as divisional headquarters of PowerCo are located in the United States. Our field work was carried out in the United Kingdom, where PowerCo operates a production site as well as the sales center for the European, Middle East, and African (EMEA) markets. PowerCo’s products are manufactured in different sizes and configurations. The largest (and most expensive) products are sold through distributors, usually in connection to a big project. They are customizable and may either be customized by the plant or the distributor. The smaller models are more standardized and sold either through distributors or directly to end customers. In the beginning of 2008, PowerCo introduced a monthly forecasting process that involved the distributors and the sales, production and supply management areas.

Our field work started in January 2009, i.e. around one year after the new forecasting process had been introduced. We established access to the case firm via a former student of one of the authors who worked as demand planning manager in the case firm. Both authors were involved in data collection and analysis. We made several visits to the field site, observed management meetings, carried out interviews and informal conversations with members of the organization and other actors, and collected various firm-internal documents. In total, we conducted 40 interviews with 29 different people between January 2009 and August 2011.^4^ One follow-up interview was conducted in December 2014. Our interviewees included finance managers, demand planning managers, production managers and business development managers within PowerCo. We also interviewed four sales managers of distributor organizations that sold PowerCo’s products. Moreover, we attended seven management meetings, which formed part of the new planning process. The meetings usually featured PowerPoint presentations, some of which we were able to obtain and use for our analysis.

As is often the case in qualitative research, our precise research focus developed during the phase of data collection and analysis. In particular, our interviews and meeting observations suggested that, while forecast accuracy was deemed an important performance measure, managers would repeatedly comment on the difficulties they encountered with this measure. This made us focus on the perceived qualities of this performance measure. We therefore systematically analyzed our interview transcripts and observation notes and looked for instances which featured either the perceived functionality of this performance measure or challenges of using it. We linked these instances to the qualities of performance indicators identified by Merchant (2006). For four out of these six qualities (timeliness, accuracy, understandability, and cost effectiveness), we did not identify any particular remarks or points of discussion among PowerCo’s managers or distributors. The remaining two qualities (goal congruence and controllability), in contrast, featured quite prominently in our empirical material. We therefore focused our empirical narrative on these two qualities. In writing up this narrative, we paid attention not to privilege the viewpoints of particular actors, but to capture the perspectives of a wide range of actors, both within PowerCo and beyond (i.e. distributors). Our particular concern was to understand why particular challenges came about and how they were addressed.

## 5. Empirical Findings

### 5.1. Background

#### 5.1.1. Planning processes

PowerCo operated production plants and sales units in different continents. The division sought to integrate its business processes through various planning and control mechanisms. Like many other large organizations, PowerCo entertained separate planning processes for different time horizons. Operational planning was done through a traditional budgeting process, established once a year for the business unit as a whole as well as for each of the sales and production units. The budget was referred to as the ‘Annual Operating Plan’ (AOP) and was supposed to be a tool for both coordination and motivation. With respect to the latter, the AOP corresponds to a certain target value for Return on Average Net Assets (ROANA), which was considered the key financial measure within PowerCo. All of PowerCo’s staff had part of their compensation linked to ROANA performance within the relevant unit. For sales and marketing staff, as well as for other support staff, ROANA was the only criterion in their bonus plans. For people in operations, half of the bonus was based on ROANA, and the other half on a set of indicators that concern conversion costs, inventory turns, delivery performance, quality, and health and safety.

The Annual Operating Plan represented a promise to shareholders and not meeting this plan would ‘raise nervousness and question marks with the investor groups’, as one finance manager put it. In the recent past, PowerCo had repeatedly either under- or overperformed compared to its forecasts, and both types of variances were considered problematic:

Now, the underperformance obviously is completely unacceptable to shareholders. But overperformance also has a set of problems that it generates for the business, not least of which is structural supply chain and causing issues for other companies and supplies. (Business Development Manager)

Against this backdrop, PowerCo introduced, at the beginning of 2008, an additional planning process in its UK plant, with the aim to better integrate the sales and operations parts of the business. The process was referred to as ‘synchronized planning’. It consisted of several steps and was carried out in a rolling manner every month. It had been designed with the help of a consulting firm. Prior to introducing synchronized planning, PowerCo relied for its short-term planning mainly on the AOP as well as on annual sales forecasts coming from its distributors. During the year, managers from different departments would come together on a rather ad hoc basis to align their activities. This practice was perceived as largely unsatisfactory, as it led to gaming behavior and the existence of different sets of numbers across the organization:

If we cast our mind back, let us say, two or three years ago, we had very much a silo mentality to planning, where you would have different people second-guessing each other. So, (…) there would be a conversation where Sales and Marketing would say they wanted 300 when really they only wanted 250, because they knew the Operations would never manufacture that … And then the people in Operations say: “No, you will never be able to do that, we would only put this in”. And this sort of hedging between departments [existed], because nobody trusted each other to execute. Because Operations would be held accountable for inventory, so if they ordered to a demand forecast that never came true, they would be held accountable for the inventory. (Finance manager)

In addition, PowerCo had seen its sales grow at unprecedented rates between 2005 and 2009, leading to an almost constant shortage in production capacities. With the economic crisis in 2009, the situation reversed, but the need to quickly react to such changes remained the same. This is particularly so since PowerCo had long lead times for sourcing the components for its products, whereas customers were demanding shorter and shorter delivery times. The new synchronized planning process was expected to help the organization better manage this tension.

#### 5.1.2. Synchronized planning

Each month, representatives of engineering, sales, production, and purchasing met to discuss the outlook and plan for the next 24 months. This cross-functional communication took place through a set of status review meetings. The first meeting in the month was dedicated to review the product portfolio and, in particular, to discuss any new product releases expected for the near future (product review meeting). This was followed by a demand review meeting in which the demand planning manager presented the sales forecast for the different product lines. This demand plan was then passed on to representatives of material planning and production who, based upon the run rates in the plant and the availability of materials, determined the volumes that they could deliver. The fourth step in the process was a meeting of demand and supply representatives, in order to agree on the production plan for the next 24 months. The joint proposal of the supply and demand representatives was then passed along to the Management Business Review (MBR) for final confirmation. Once the MBR had confirmed the numbers, the forecast could be loaded onto the production system and, from then on, became consequential for the plant. The resulting forecast was referred to as a ‘constrained’ forecast, since the negotiated forecast between demand, production and supply was constrained by supply and production capacities and thus did not fully reflect the potentially greater ‘unconstrained’ market demand.

The second step within the synchronized planning process, i.e. the demand review, relied to an important extent on input provided by PowerCo’s distributors. The dense distribution network that PowerCo maintained was one of the competitive strengths of the firm. PowerCo’s contact with these distributors was through Business Development Managers (BDMs) who acted as sales coordinators for particular regions or countries. Each month, distributors would send their sales forecast numbers in a spread sheet format to their BDM who might modify these numbers before passing them on to the Regional General Manager (RGM) and the demand planning manager. In a ‘consensus meeting’, the RGMs and the demand planning manager considered the aggregate demand information, potentially modified the overall forecast, and decided on the final demand plan to be presented in the demand review meeting.

In addition to the monthly held meetings, PowerCo introduced a set of performance measures (‘elementary eight’)^5^ in order to assess how well the synchronized planning cycle was operating. The use of these performance measures was recommended by the consulting firm that advertised them as mechanisms assessing and furthering a ‘capable level’ of planning and coordination along what they called a ‘maturity journey’ of business performance. The indicators should help achieve capable planning by indicating possibilities for improvements. Importantly, they should also promote honest information exchange along the supply chain by instilling ‘confidence’ in the joint planning process and by increasing accountability of all involved parties (see training documents/presentation slides). All eight indicators were defined as measures of accuracy. There was, for example, a measure for the accuracy of the bill of materials and another one for the accuracy of customer delivery times. These indicators were formulated as percentages and the target level for most of them was set at 95%.

The consulting firm put forward a rationale of highly interrelated indicators: the accuracy of one indicator was seen to depend on the accuracy of (at least some of) the other indicators. However, in order to practically deal with these indicators, responsibilities for the eight indicators were separated. For each indicator, one manager was given the responsibility to lead a task team that would come up with action plans in order to improve the performance on the indicator. Progress on these actions plans was reported regularly in specifically instituted meetings. The indicator of forecast accuracy was seen as particularly crucial in order to achieve better integration and coordination along the supply chain.

#### 5.1.3. Forecast accuracy

In order to assess the quality of demand forecasting, PowerCo initially introduced two forecast accuracy indicators, one for a six months period and the other one for a three months period. In each case, forecast error was defined as the absolute value of the difference between actual sales (units) and forecast sales (units), divided by the actual sales. The inverse of this was the measure of forecast accuracy. Forecast accuracy was calculated separately for each production line as well as for the plant as a whole, on the basis of a mean absolute percentage error (MAPE).

After the implementation of the forecast accuracy indicators in 2008, levels of forecast accuracy were hardly satisfactory. The values of the two indicators were far from the 95% that the consulting firm had suggested as a target. The values also fluctuated considerably from month to month, and the reasons for these fluctuations were not always understood. What was known was that inaccuracy might be generated at different levels within the decision chain. Distributors submitted their numbers to Business Development Managers, who could make changes to these numbers before submitting the aggregate forecast for their distribution area to the Regional General Managers and the demand planning manager, who then aggregate the numbers for the synchronized planning review meetings. Within this chain, the input provided by the distributors was considered particularly crucial. Accordingly, management decided to develop a separate measure of forecast accuracy that could be used to specifically monitor the quality of the distributors’ input. This indicator was implemented in spring 2011. The rationale was to increase distributors’ forecasting skills and their motivation to provide accurate sales forecasts:

The main idea is that we get forecasts each month coming in from the distributors and the problem is that if they don’t forecast accurately, this affects the supply chain […] the idea is to sort of try to improve their accuracy of forecasting by showing them how accurate they were in previous months. (Marketing Support Analyst)

In order to hold distributors accountable for their forecast accuracy, an indicator was developed that should capture the *unconstrained* forecast accuracy which could be ascribed exclusively to distributors and not to other parties in the supply chain. Therefore, the forecast accuracy measure for distributors was derived from the distributors’ forecast orders as compared to orders by customer request date, while the overall (constrained) forecast accuracy measure was based on the constrained forecast resulting from the synchronized planning process as compared to orders by promised date, i.e. as promised by the plant. The performance measure was calculated as the mean of four different forecast accuracies, i.e. for 3, 4, 5, and 6 months out.

We look at the demand plan 3–6 months out, because this is the time in which the supply chain can respond to change in demand. I have noticed actually, when we have done analysis of forecast accuracy in the plant, at 6 months out, we show a level of what we have been forecasting and sometimes, they are quite in line with the actuals we have done, although this is not showing truly the accuracy that we have achieved, because 3 months out, we have had a lot more demand come in, but this is demand that supply has not been able to meet, so the actuals have actually been closer to the 6 months out, because this is what the supply chain has been able to produce, whereas when it gets to 3 months, this is where the supply chain struggles to try and meet the demand. (Marketing Support Analyst)

**Figure 1.**
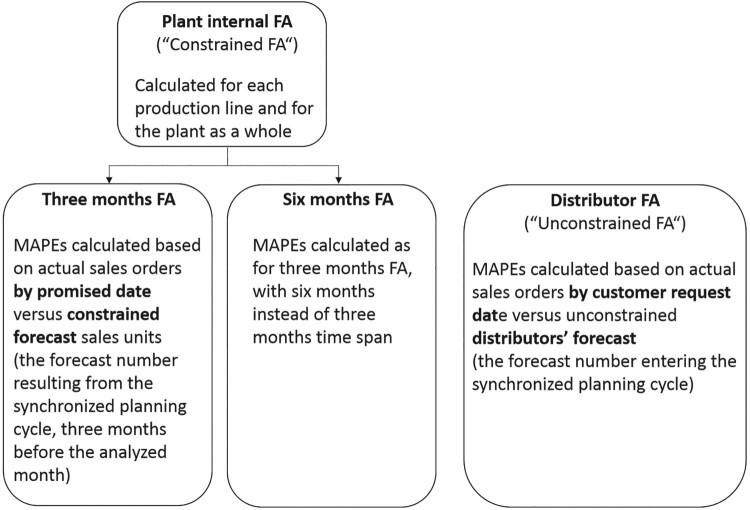
Three types of forecast accuracy measures used in PowerCo.

In the short run, distributor forecast accuracy was included as an indicator in a one-off sales initiative, called ‘Going for Gold’^6^ which was introduced at the beginning of 2011. This initiative should incentivize distributors to try and make increased sales efforts as well as to forecast accurately. Distributors competed in ‘Going for Gold’ on four indicators: total sales, sales in a particular industry segment, sales in one particular product category, and distributor forecast accuracy. In the long run, the idea was to include distributor forecast accuracy as one of the performance indicators against which distributors would be regularly measured. Figure 1 displays the three different types of forecast accuracy measures that PowerCo implemented during the studied period.

From the above description, we can identify several circumstances that allowed forecast accuracy to gain visibility and importance within PowerCo. First, forecast accuracy was part of the ‘elementary eight’ performance measures which PowerCo had put at the center of their operational activities. Not only did forecast accuracy therefore benefit from the legitimacy of the other elementary performance measures; there was also the clear acknowledgement of interdependencies between these measures which made it important to pay attention to forecast accuracy. Without a reasonable quality in forecasting, excellence in the other operational domains was considered difficult. Forecast accuracy was thus enacted as goal congruent with other objectives considered important (Merchant, 2006). Second, forecast accuracy was embedded in the new synchronized planning process which was argued to be a fundamental activity for PowerCo. Within the regularly held planning meetings, forecast accuracy was repeatedly reported and discussed and was thereby enacted as an important calculation (Jordan & Messner, 2012). The training sessions that the consulting firm had organized for the synchronized planning process added to this relevance of forecast accuracy. Third, with the creation of a separate indicator for distributors’ forecast accuracy, PowerCo had established clear accountability for distributors. The particular importance of their input was recognized through the ‘Going for Gold’ initiative, whereby distributors were given incentives to improve their forecast accuracy.

Notwithstanding these initiatives and circumstances, however, there were concerns with the use of forecast accuracy as a performance measure. Forecast accuracy was considered a somewhat problematic performance measure mainly for two reasons. On the one hand, there were concerns that distributors could not sufficiently control forecast accuracy and that tightening results control in this respect might be insufficient to improve the quality of the forecasts. On the other hand, there were also questions about the desirability of an increased focus on forecast accuracy. While from a plant’s perspective, accurate forecasts were considered very important, the distributors and sales part of the organization had different priorities that were in a sense competing with forecast accuracy for managerial attention. The following section elaborates in more detail on these two types of concerns and on how PowerCo responded to them.

### 5.2. Concerns with the use of Forecast Accuracy as a Results Control

#### 5.2.1. Is forecast accuracy sufficiently controllable?

The task of the distributors was to indicate the ‘unconstrained’ demand that they were expecting for the coming 12 months. This was a challenging task, even for those distributors that dedicated a lot of time and effort to get their forecasts right. Demand patterns were hardly stable in most countries and distributors had difficulties in predicting how much they would be able to sell. This lack of controllability over demand was very much emphasized by distributors’ sales managers when they referred to the particular economic and political situation in their respective markets. A case in point is the unexpected crisis brought about by the ‘Arabic spring revolutions’ of 2011 in the Middle East:

Look, these exceptional days in Egypt nowadays, we never had this before, because it is the first time with these troubles in Egypt for I think more than 50 years. In our life, we didn’t see it. So, it is very difficult and no one, even if I can say we are doing a good forecast, but no one in these days can make a correct forecast. (Sales manager of Egyptian distributor)

However, not only such exceptional events appeared to make forecasting difficult. Also more everyday conditions, such as ‘the trading culture’ in a particular geographic region that might be characterized by short-term deals rather than long-term planning play an important role:

If you consider for example Russia, where in general there is not so much business planning, and there are a lot of businesses which are very much reliant on last minute decisions on whether you get it or not. (Demand planning manager).

Similarly, some countries were subject to frequent regulatory changes that impacted the level of business. For instance, the BDM responsible for Pakistan explained the impact of a recent tax change in Pakistan:

There will be more subtle changes going on within a particular country that could impact [actual orders]. Classic example would be Pakistan. The government recently in a period of two weeks changed the duty from 5% to 17% on certain [products]. Now that’s going to have an impact. Whilst it’s applicable to everybody, it’s going to maybe turn some customers off that were planning on doing something. (BDM responsible for Pakistan)

As these examples illustrate, controllability of forecast accuracy hinges on the relative (un)certainty of demand. The more uncertain the demand, the less appropriate it will be to manage the forecasting process mainly by using forecast accuracy as a results measure to control distributors ‘at a distance’. Calling forecast accuracy a ‘truth-inducing’ measure (e.g. Brüggen & Luft, 2011; Chow et al., 1994; Waller, 1988) then also seems problematic, given that the problem is not only (or primarily) that distributors don’t want to tell the truth, but that the ‘truth’ is basically unknown to them.

The problem of controllability was partly also an internal one, in the sense that those managers within the distributor organization who were responsible for demand planning did not always have the ‘last word’ on the units that would be planned for. Indeed, in some distributor organizations, sales managers had to get their forecasts approved by their finance departments. A BDM explained to us that, sometimes, the volumes requested by the commercial department were not the ones that would ultimately be approved by the finance department of the distributor, thus causing difficulties for PowerCo to understand the underlying market demand:

We get the demand planner from the commercial department, the [divisional] leaders, the heads of the people selling. If he wants something and the finance person doesn’t place it, and he puts 10, and he says no, then that throws the whole process … you’ve got to really make sure that you’ve got reasonable alignment within that organization, and sometimes we have to bring the two together. But as they are fairly big organizations, bring these two department heads together, and say, look guys, we need data to come from one person, it’s no point [sales] saying, I need 10 sets and you, finance, won’t put 10 sets in stock, it defeats the objective. It just makes us inaccurate, so we need to understand, if you’re not comfortable with what he is doing, then what level of inventory or stock are you comfortable with, and then get the agreement and then work on that basis. If it’s slightly lower, then at least we get accuracy. But we don’t want this lack of agreement within one company. (BDM)

In addition, there was also a conceptual issue with measuring forecast accuracy that introduced a problem of controllability. Recall that distributors’ forecast accuracy was based on ‘unconstrained’ demand, in the sense that forecast orders were compared with orders as *placed* by customers, rather than with orders actually fulfilled. This was deemed important in order to avoid that supply constraints would impact distributors’ sales accuracy. However, the influence of supply constraints could not be completely eliminated. This was because it was known that customers would sometimes anticipate delivery problems on the part of PowerCo (i.e. unavailability of the product or long lead times) and, as a consequence, would decide not to place an order (even though these orders were correctly forecast). In fact, there was an established practice of informally inquiring with the Customer Service Department and then deciding on whether to place an order or not:

So, most of the unconstrained demand was typically simply discussed on the phone between the distributor and the Customer Service with a simple … simple answer … simple question: “We have got a customer asking for 50 units in the next three weeks. Are we able to produce them in the next three weeks?” – “No, we are not.” – “Okay.” (Demand planning manager).

This resulted in so-called ‘lost sales’ and these lost sales obviously negatively impacted distributors’ forecast accuracy. Making distributors accountable for accurate sales forecasts was therefore problematic as long as there were supply constraints, because distributors could always argue that these supply constraints resulted in lost sales compared to the correctly forecast sales volumes. As the demand planning manager put it:

This measure is not driving the improvement, because there is no point in me going out to the sales team and to the distributors, saying that they are forecasting inaccurately, when part of the reason of the inaccuracy is not due to their forecast, but to supply constraint.

The use of forecast accuracy to measure and incentivize distributors requires separating the activities on the demand side from those on the supply side. If this is not possible, as in our case, concerns with the validity of this measure are likely to emerge.

Related to these problems with distributor controllability over forecast accuracy, it was a non-trivial task for demand planning to specify target levels of forecast accuracy for distributors. Clearly, the 95% accuracy target specified by the consultants was impossible to achieve in the short and medium run. For motivation and evaluation purposes such unrealistic targets were increasingly regarded as of limited use. Thus, the question arose what could be a more useful target to be communicated to distributors:

And today, the issue is, especially, when we speak about customers, we go to the distributors and say, ‘Look, here is your Forecast Accuracy.’ We tell them, ‘Look, you have got an error, which is 50 per cent; only 50 per cent of the Forecast Accuracy.’ They come back to us saying, ‘Okay, but where can I get? I cannot get 200 per cent forecast accuracy. What is the level actually?’ (Demand planning manager)

The demand planning manager would have liked to have industry benchmarks in order to be able to better argue for a certain level of accuracy, but such benchmarks were not available to the firm. He also acknowledged that having the same targets for all distributors was somewhat problematic, given that forecast accuracy was sensitive to volume. A low amount of absolute units ordered (volume) went along with a high potential variance in accuracy:

[…] The other problem is the fact that this target should change a lot depending on volume of business. Sometimes, distributors have got too low quantities for specific models and ultimately, you end up with [inaccuracy]. So, while this overall target makes sense on the regional level, when it comes to the distributor level, it is very complex. (Demand planning manager)

To summarize, we can see that various situational factors and conditions challenged the controllability of the forecast accuracy measure. Partly these were located in the market and were thus external to the organization; partly they resulted from specific practices prevalent in PowerCo and its distributors. Both types of controllability problems limited the usefulness of the forecast accuracy measure as an instrument for results control. In particular the controllability issues related to intra- and inter-organizational practices highlight the specific nature of forecasting in business organizations where outcomes of sales, costs and profits are not simply external factors to be forecast correctly (similar to a weather forecast), but are usually also targets and organizational actors can actively do things in order to influence the outcome. Sales planning accuracy then is a function not only of the distributors’ planning skills, but also of the ability of the wider inter-organizational network to realize the plans. As our case shows, interdependencies between demand, production and supply, reinforced by specific practices such as the cross-consumption between demand classes, significantly limit the distributors’ controllability over, and their willingness to be accountable for, the forecast accuracy results.

#### 5.2.2. Is a strong emphasis on forecast accuracy desirable?

Besides a lack of knowledge of, and influence over, future demand, there were also motivational problems involved in using forecast accuracy as a performance indicator for distributors. It was apparently challenging to convince distributors of the importance of diligent and time-consuming forecasting processes. As one manager put it, ‘it’s hard to convince sales people necessarily that that’s a good use of their time’.

In order to improve distributors’ forecasting accuracy, PowerCo deemed it necessary to show to the distributors that there was a benefit for them if they forecast accurately. Potential benefits for distributors would be decreased lead times and assured availability of the forecast demand. However, it could not be clearly shown to which extent accurate forecasts actually led to these promised benefits. This was so because improved forecast accuracy was not the only factor impacting availability and lead times. The overall demand level, constraints caused by suppliers and the plants’ stock policy played an important role in this regard:

They [distributors] invest time every month, they do the work, they want to have a return. Now that we also measure the work, they will come back to us probably even with more wish, urgency and pressure to seeing a return. The return that we have to give is improved lead times. This is the only thing they mind. This is the main reason for which we ask them to give us a forecast, and we put in place the whole demand planning and synchronized planning process. So what would be great would be to show the correlation that we’ve got between good forecast accuracy and improved lead times. And something that we have not been able to do is exactly showing this correlation. We are not able to do it first of all because of several supply constraints […] If customers start perceiving that actually you are continuing having these supply constraints, what’s the point in me giving you the forecast. We will lose their interest. (Demand planning manager)

How forecast accuracy, lead times, stock policy and supply constraints were interrelated was not fully understood. Some believed that forecast accuracy and availability/lead times did not correlate because distributors’ forecasts were not always taken sufficiently ‘seriously’ at plant level. This meant that timely forecasts would not always ensure availability of the forecast product, even if the order came in as forecast. As a master scheduler put it,

I think that unfortunately over time, there may be a perception from people that are providing us this vital information, that we do not take it that seriously; therefore, they do not take it so seriously and possibly, we are getting mixed signals.

Failure to always ensure timely delivery of correctly forecast products was related to the practice of ‘cross-consumption’ across different demand classes. A demand class comprised a group of customers, typically grouped by region. PowerCo had introduced this concept in order to create a link between forecast and actual orders. If forecast orders were consumed by actual orders only *within* (and not across) demand classes, distributors for a particular region would receive products when they had correctly forecast them. As such, demand classes were regarded as important to create accountability for forecasts and to credit those distributors who forecast accurately. At some point, the use of demand classes was effectively abandoned, however, as PowerCo started to allow for ‘consumption’ of forecasts across different demand classes. That is, a customer would be served if there was an ‘available’ forecast order in the system (and thus a product produced to that forecast), even if the forecast did not come from the same customer (or from within the customer’s region).

At the time of our research, there was an increased sense that this practice of cross-consumption was problematic because it did not generate incentives for distributors to deliver more accurate forecasts. It also created particular challenges for small customers, who were not able to place firm orders as easily as large customers:

Small distributors were not able to commit buying in advance, because they don’t have stock facilities, they don’t have the financial availability. They need to have the end user ordering to them before they can order it to the plant. As a consequence of the fact that they were not able to place orders in advance, that was causing them to always suffer from restricted availability, especially in comparison to the larger distributors who were able to speculate and placing orders in advance. In order to offset this behaviour we were saying we want to get rid ideally of the principle first come – first served, we want to be able to offer better lead times to those customers or group of customers which are accurate in their plan. Sort of recognising that their forecast accuracy improves our plan and use this as a rule for assigning units. (Demand planning manager)

Yet, re-implementing the concept of demand classes met with technical limitations of the new Oracle tool. Moreover, the case for using such demand classes was also contested. After all, cross-consumption of orders was a more efficient way to match demand and supply (as all available forecast orders would be consumed) and was, on average, better for satisfying customers.

Another set of motivational challenges concerned potential incentive conflicts caused by existing performance indicators that were perceived to work against the forecast accuracy indicator. Such conflicts were seen to exist particularly on the level of distributors and the Business Development Managers (BDMs). BDMs were the main point of contact between the plants and the distributors. Motivating distributors to dedicate resources to forecasting, therefore, hinged to a large extent on these BDMs. The performance of BDMs, however, was measured mainly against sales targets as agreed upon during the annual budgeting process (AOP). BDMs were not particularly incentivised to promote forecast accuracy:

And I think part of the problem is, when we do the forecasting, they [distributors] take a lot of input from BDMs who are actually in the field in the various countries. I don’t think those BDMs necessarily take ownership for the inventory that we have here, because they don’t see it. For them it’s always about hitting the revenue number. But from my perspective, you’ve got to hit the revenue, but you’ve got to hit it on the right timeframe. It’s not helpful to me that you are predicting 50 million dollars each month, then I have nothing in one and 100 million the next, you hit your number, but I am completely out of sync, because I have tons of inventory. (Supply manager)

This challenge was linked to performance metrics more generally. Sales people were measured on margins (ROANA) only, while plant people were mostly measured on plant measures, including inventory turns. Some managers on the plant described this measurement system as a cause of ‘disconnect’ between the functions. Even though the ROANA indicator included inventory as a part of net assets, supply managers argued that inventory would count far less for sales managers as compared to supply managers. BDMs and RGMs would therefore be incentivised to over-exceed their annual sales plans in terms of revenue figures, rather than meeting monthly sales forecasts in terms of product units. As a finance manager put it, ‘they are measured predominantly on gross margin and that type of things (…) and even over-exceeding the plan, they would probably get much more favorable than missing it, whereas for us, variance is absolute.’

For distributors, the most important control tool was the DAOP. The DAOP was the annual target that served as a basis for renewal of contracts with distributors. The monthly forecasts, in contrast, were supposed to be the realistic expectations about the market demand:

Because the DAOP is a document that has got a list, key strategic initiatives that the distributor is going to take for the following year, but it has got also a plan, in terms of how many units for every [product] model they are targeting to sell for the following year. So there is a moment in time in which this plan should be consistent with the forecast, with the demand plan that they submit. Then as we move forward, the target remains fixed, but the forecast, the demand plan every month can change. […] It is getting more realistic, closer to what the actual demand will be, hopefully. (Demand planning manager)

Forecast accuracy as a performance indicator was in conflict with DAOP targets such as sales and market share, since ‘outperforming’ was good in DAOP terms, but bad with regard to forecast accuracy. For distributors, hitting their DAOP targets was perceived to be significantly more relevant than achieving high forecast accuracy:

Demand planner accuracy is one criterion, ok, the other thing, the other reality is the number of units you have sold. So if DAOP is 3 million, maybe we are 30% accurate, but if we have achieved the 3 million DAOP, so finally the goal is achieved, that’s it. (…) apart from this demand planner accuracy, there are other things required like the organizational structure, the market share, like if we are sharing 30% of the market – obviously we are not sharing this right now – but if we are having 30% of the market share. So, evidently, our demand planning is not that good, but we are sharing very, very good market, so in my view, this is not the only target. There are some other targets, so you have to include these. (Sales manager of an independent distributor organization in Pakistan)

Besides a lack of motivation to work towards forecast accuracy targets, the strong focus on revenue targets was also believed to influence and therefore bias the forecasting process itself. That is, distributors, BDMs and RGMs were suspected by plant managers to manage the forecast towards the DAOP and AOP targets rather than basing the forecast on the current market situation:

In [PowerCo] we are so driven by yearly behaviour, by hitting our AOP numbers. That, even if SP is telling you, say for example AOP is set at 100 million dollars in revenue and synchronised process is telling you 60 million will be your revenue, the demand people will forecast 100 even though they can’t sell. Because AOP is 100 and they don’t want to get asked questions on why you are missing your AOP number. (Supply manager)

For BDMs, the DAOP was a natural point of comparison for the forecasts, so that not only current market situation, order status and trends would influence the forecast, but that they would check their distributors’ forecasts as to their alignment with the DAOP:

Well, obviously, we’ve set a business plan of x number of million, and if the demand plan comes in, and in total of units it says they are going to do 30% of that, then my job as a business development manager is to understand how do we close that gap. I can’t just go on and say, oh, fine, I’m happy with you doing 30% of what we agreed you’d to, that’s no good. So we work with them to say, well, why is there a gap? Do you realise there is a gap? And probably sometimes they go and get lots of small [products] and it looks very impressive, but of course when you get to the bigger [products] which actually make the dollars in terms of a plan in revenue and income to the corporation, it’s not there. (…) They (distributors) need to be mindful of those goals, because what’s the point of us agreeing a plan and then (…) them actually sending us forecasts in, saying they are going to do 30% of what they’ve predicted? … The challenge is though, I think you also need to be mindful, so they don’t use the demand planner to what we call “feed the monkey”, give me the answer I want to hear. Saying, no, those [products] add up to their budgets at the end of the year, and they don’t really do it. So I go, “Oh, lovely, it says it”. So we have to be mindful of that. (BDM)

Since forecasts were influenced by the goals as defined through the DAOP, the forecasts did not necessarily reflect the ‘most honest’ estimate of future demand (Hope & Fraser, 2003). Having a performance measure for forecast accuracy should help avoid exactly such behavior (Brüggen & Luft, 2011; Chow et al., 1994), but apparently the pressure to ‘match the DAOP’ was higher than the incentive to provide a completely ‘honest’ forecast. Hence, in this case, the characterization of forecast accuracy as a ‘truth-inducing’ performance measure (Brüggen & Luft, 2011; Chow et al., 1994) is challenged not so much by problems of controllability (see above), but by competing goals (in this case the objective of achieving or even outperforming the annual budgeted sales target vs. the objective of achieving sales forecast accuracy).

#### 5.2.3. Responding to concerns

The above outlined challenges were present throughout our observation period. Yet, we could observe that, over time, PowerCo developed some initiatives to control the forecasting process that went beyond a pure focus on measuring the level of accuracy.

One of these was to simplify the information transmitted to distributors in order to avoid cumbersome discussions about the ‘correct’ measurement (and aspiration level) of forecast accuracy. In particular, indicating whether distributors were continuously over- or under-forecasting made it easier for PowerCo to argue that there was a problem with the forecast:

What I like is the fact that more recently we are showing the [forecast accuracy] measure, but also just highlighting whether there is an over-forecasting or under-forecasting bias, which I think is very good, because this is something that you can easily measure and as a sales person who submits the forecast, you cannot say: ‘No, it’s not right’. Or you cannot find an excuse. In the case, in which consistently, for 4, 5, 6 months, the measure is showing that you have been over-forecasting, then there is definitely something you need to do in terms of how you forecast. You know that, next time, you need to work out the same forecast as before, but then reduce it for some level, because history is telling you that you are over-forecasting. (…) It is very easy to understand and it is also easy to take action, to fix it. (Demand planning manager)

In addition, there were increased efforts to ‘educate’ distributors regarding the importance of forecasting accurately. Communicating merely the results was deemed insufficient in this respect; a more hands-on engagement with the distributors was necessary. In the below example, a Business Development Manager explains that it would be necessary to communicate not just with the sales leader of the distributor, but also with the people who ‘feed the data’:

One of the [tasks of a BDM] is actually making sure that the sales staff, our distributors, are fully appreciative of this process. I was recently in Pakistan, and normally we deal with the sales leaders at the distributor, we work with them and we give them an overview of why this is an important process, but if people who feed the data to them, who then feed the data to us, if they don’t understand the importance of the process, how it works, then you get another reason for a possible error. And so we found, it was quite interesting, we had I think 15, 20 sales men from Pakistan and I actually showed them the actual error … I think one piece of data said they were 900% inaccurate on their forecast of this particular model. Now I said, would you be happy being 900% inaccurate on your wages? So … they wouldn’t and after a good hour’s discussion, they actually started to understand the real need for them, even though they are lower down within the organization, the need for them to understand and give quality data as best they can to their superiors who then feed it to ourselves, and then it goes on to produce an outcome. (Business Development Manager)

The demand planning manager confirmed that there was an increased focus on engaging in a close dialogue with the distributors so as to discuss in detail their *inputs* to the planning process:

Yes, and the idea of this has moved away from: ‘OK, we received your forecast, thanks, can you please give us additional qualitative information’, so this was the setup at the very beginning. Now it’s more: ‘OK, let’s discuss together’. There are a lot of questions to the distributors which challenge the number and, ultimately, we very often have as end result that this forecast discussion has some action items for the distributors to review numbers of specific models and resubmit the numbers according to additional information or a review of the forecast accuracy or re-discussing of the confidence level of some numbers.

These initiatives indicate a shift in PowerCo from enacting forecast accuracy primarily as an issue of revealing one actor group’s (the distributors’) private information towards understanding forecast accuracy as a cooperative achievement that is influenced by interactions within and across the involved organizations.

## 6. Discussion

In this paper, we have drawn upon a single case study to examine the use of forecast accuracy as a performance measure. Our paper is, to the best of our knowledge, the first one to empirically document managers’ experiences with using forecast accuracy as a management control tool. In particular, we show how the case organization, PowerCo, implemented a performance measure for forecast accuracy as part of its initiative to improve the quality of their operational plans. The paper is informative about two related issues. First, it offers insights into the use of a forecast accuracy indicator as *one* way of trying to improve the quality of planning. Second, it sheds light on the challenges in achieving high levels of forecast accuracy and problematizes the notion of a ‘truth-inducing’ measure.

### 6.1. Forecast Accuracy Indicator as a Complement to Interactive Forms of Planning

Our case shows how a performance measure for forecast accuracy can be a helpful complement to other ways of trying to improve the quality of planning. Indeed, prior empirical literature suggests that firms can address problems of budgeting by introducing more interactive (‘dynamic’) forms of planning. Bourmistrov and Kaarbøe (2013), for instance, examine the application of ‘beyond budgeting’ ideas in two large firms, an oil firm and a telecom firm. In both firms, there was a ‘growing recognition that the necessary information for making relevant decisions in the context of continuously changing markets was not provided by the budget’ (p. 202). The authors observe how the introduction of rolling forecasts created a climate in which more frequent discussions about gaps between forecasts and targets were held. ‘In both organizations, the realistic forecasts were updated continuously, which resulted in better opportunities to learn from changes in the environment and thereby seize new business opportunities’ (p. 207). Similar observations have been made in other studies (e.g. Goretzki & Messner, 2016; Henttu-Aho & Järvinen, 2013; Østergren & Stensaker, 2010). Our case company also believed in the importance of such interactive forms of planning. The synchronized planning process that was introduced with the help of a consulting firm was mainly about installing a set of regular meetings in which assumptions should be challenged and decisions taken in a cooperative spirit. At the same time, our case differs from those reported in prior literature insofar as our case company complemented the interactive forms of planning with a results control measure for forecast accuracy.

The promise of such a results control mechanism is to manage distributors ‘from a distance’. It is obviously much less resource-intensive than having a highly interactive process with each of the distributors, and it provides quantified information on an important part of the planning process, i.e. sales forecasting. Together with other performance measures (the ‘elementary eight’ in the case of PowerCo), a forecast accuracy indicator can therefore be a useful complement to more interactive forms of planning as it indicates how much progress a firm makes with its efforts to better align the demand and supply side.

Yet, realizing this potential of forecast accuracy can meet with challenges. We could observe in our case company concerns with the *limited controllability* of the performance measure as well as with competing interests that challenged the perceived *goal congruence* of forecast accuracy. It is important to document such challenges given that the accounting literature has so far mainly pointed to the potential benefits of forecast accuracy in terms of alleviating problems of misreporting (e.g. Brüggen & Luft, 2011; Chow et al., 1994; Waller, 1988). Our paper by no means denies these benefits, but cautions that realizing them may be quite challenging in practice. It is here where the *situated* functionality (Ahrens & Chapman, 2007) of performance measures comes into play. How and in which context forecast accuracy is mobilized matters for the effects that this measure will have in an organization (see next section).

We could observe that the challenges with forecast accuracy resulted in efforts to simplify the communication of forecast accuracy information as well as to more closely interact with distributors and to move beyond a focus on results control. This change in the approach to forecast accuracy helped seeing the performance measure as a learning tool (Davis & Mentzer, 2007) that can trigger efforts to improve the forecasting process. Since PowerCo started to implement the more cooperative approach to forecasting only at the end of our study period of 2.5 years, we cannot say to what extent this approach systematically improved the forecast accuracy results. In a recent study, Glaum, Schmidt, and Schnürer (2018) illustrate how cash flow forecasts at Bayer became more accurate when those who provided the forecasts and those who received them interacted more closely in order to review input data and learn to interpret exceptional cases. More research is required in order to more fully understand the ways in which interactive forecasting practices, and specifically the interactive use of forecast accuracy indicators, relate to the quality of planning in specific contexts.

### 6.2. On the Notion of a ‘truth-inducing’ Measure

The literature often refers to forecast accuracy as a ‘truth-inducing’ performance measure (e.g. Brüggen & Luft, 2011; Chow et al., 1994; Waller, 1988), the underlying idea being that actors have an incentive to report the truth when being measured on forecast accuracy. Our empirical observations suggest some caution regarding this idea. First, it shows that incentives for forecast accuracy are at times not strong enough to compete with other concerns, such as showing progress towards reaching the annual budget numbers (DAOP). The practice of ‘matching’ the forecast to the target that we could observe in our case firm is a form of gaming behavior (Lukka, 1988; Merchant, 1985), but one that is not motivated by the intention to create budgetary slack, but rather by actors’ desire to signal commitment to the budgetary targets. Using a performance measure for forecast accuracy can, in principle, alleviate this problem, but only if the incentive for accurate forecasting is sufficiently high. In the case of PowerCo, the incentive was apparently too small compared to the felt pressure to ‘signal commitment’ to the targets when forecasting during the year. Moreover, it was not obvious to distributors that they would benefit from more accurate forecasting. PowerCo struggled to provide evidence on positive consequences such as improved lead times, and this was not least due to the particular way of consuming customer orders (i.e. cross-consumption between demand classes).

Second, and more fundamentally, our study evidences problems of controllability that may emerge with the forecast accuracy measure. Forecast accuracy is a rather specific type of performance measure that requires controllability on two levels: regarding the activity of forecasting sales and regarding the activity of realizing the forecast sales. A high level of uncertainty in demand poses a challenge in both respects and makes it more likely that forecast numbers will differ from realized ones. This was indeed one of the major concerns reported by PowerCo’s distributors, who faced uncertain market environments and therefore struggled to come up with accurate forecasts. Moreover, our study highlights uncontrollability that arises from the specific relations within and between inter-organizational actors. Within distributor organizations, interdepartmental relations were relevant in this regard, as the power of the finance department to override the sales forecast limited the sales department’s controllability over the forecast. Specific inter-organizational interactions between distributors and PowerCo also accounted for the distributors’ limited controllability over forecast accuracy results, as PowerCo’s supply constraints and their practices of cross-consuming orders between demand classes impacted the distributors’ forecast accuracy.

Our observations regarding goal congruence and controllability of forecast accuracy measures challenge the terminology and the often taken-for-granted assumptions of ‘truth-inducing’ indicators in the literature (i.e. that achieving high performance on these indicators is a matter of ‘choosing to tell the truth’, and that these indicators are per se goal congruent). As other scholars have pointed out, the typically proposed interventions to ‘induce truth’ by means of results control measures are based on the problematic assumption of agents’ general propensity to lie (Evans et al., 2001; Salterio & Webb, 2006). We would add to this criticism that the quality of planning is not necessarily a problem of revealing private information (i.e. choosing to tell the truth or to lie), but rather a problem of getting the involved actors to cooperate in order to better understand mutual interdependencies and constraints in the planning process. As our case study illustrates, the assumption of clear-cut hierarchical relations between agents and principals, an assumption that typically underlies experimental studies on truth-inducing indicators and budgetary slack, is not particularly helpful in order to understand more complex organizational and inter-organizational dynamics, where several hierarchies, actors and accountabilities interrelate. For instance, we have seen that the distributor organizations cannot be regarded as uniform ‘agents’ vis-à-vis the ‘principal’ of PowerCo, since the distributors’ demand planners depend on, and may be in disagreement with, the decisions of the distributors’ finance departments. Likewise, it is problematic to envision PowerCo in terms of a uniform ‘principal’, given the partly conflictual relations between sales, production and supply departments as well as the particular practices of how to consume customer orders *within* PowerCo.

## 7. Conclusion

We have focused in this paper on one particular performance measure, i.e. forecast accuracy, and its relevance for improving the quality of firms’ planning processes. Of course, firms use many different performance metrics and forecast accuracy is obviously just one of them. While prior literature has indeed zoomed in on other specific indicators such as customer satisfaction (Ittner & Larcker, 1998), return on investment (Swieringa & Weick, 1987) or Economic Value Added™ (Mouritsen, 1998), it has not been our interest in this paper to study forecast accuracy simply for the sake of extending this list. Rather, our study is motivated by a long-standing concern with the quality of firms’ planning practices (e.g. Lukka, 1988; Merchant, 1985; Shields & Young, 1993), which has recently gained momentum as researchers have become interested in firms’ use of rolling forecasts and the application of beyond budgeting ideas (e.g. Becker, 2014; Bourmistrov & Kaarbøe, 2013; Libby & Lindsay, 2010). Forecast accuracy represents *one* way of addressing concerns with planning quality. While the accounting literature mentions the potential of forecast accuracy to address problems of gaming behavior (e.g. Brüggen & Luft, 2011; Chow et al., 1994; Waller, 1988), we lack empirical evidence on firms’ actual use of this performance measure. Our study offers such an empirical investigation and shows how forecast accuracy can become subject to discussion and debate, as concerns with goal congruence and controllability render it an imperfect measure. Our analysis highlights the specific conditions under which these concerns arose in the case company and the ways in which the case actors sought to deal with these issues by adapting their forecast accuracy practices towards more interactive forms of performance management. Viewing the qualities of performance indicators as fundamentally situated calls for complementary case studies in other contexts in order to understand more fully the different ways in which forecast accuracy measures are enacted.

## Footnotes

1 For reasons of confidentiality, the name of the organization has been rendered anonymous.

2 Chen, Rennekamp, and Zhou (2015) is an exception insofar as they examine possible psychological biases in forecasting one’s performance.

3 Challenges may of course emerge also for reasons not related to the qualities of performance measures. However, since our interest here is in improving our understanding of one particular measure, i.e. forecast accuracy, we decide to focus on such qualities.

4 About half of the interviews were carried out by both researchers together. Five of the interviews were carried out by a research assistant.

5 The ‘elementary eight’ performance measures comprised bill of material (BoM) accuracy, routing accuracy, inventory record accuracy, new product launch adherence, forecast accuracy, supplier delivery performance, on-time-production, and customer delivery performance.

6 The title of this initiative reflects the prize that was given to the best performers, i.e., tickets for attending part of the Olympic Games in London.
